# Percutaneous Venous Cannulation for Minimally Invasive Cardiac Surgery: The Safest and Effective Technique Described Step-by-Step

**DOI:** 10.3389/fsurg.2022.828772

**Published:** 2022-03-22

**Authors:** Alberto Pozzoli, Tiziano Torre, Francesca Toto, Thomas Theologou, Enrico Ferrari, Stefanos Demertzis

**Affiliations:** ^1^Division of Cardiac Surgery, Cardiocentro Ticino Institute, EOC, Lugano, Switzerland; ^2^Faculty of Biomedical Sciences, Università della Svizzera Italiana (USI), Lugano, Switzerland

**Keywords:** minimally invasive cardiac surgery, venous cannulation, echo-guided venous puncture, wire skills, transesophageal echocardiography

## Abstract

The current cardiac surgical landscape, with the expansion of minimally invasive operations, ECMO, and some interventional therapies, requires a thorough knowledge of peripheral cannulation techniques. In particular, venous cannulation may appear trivial and complication-free, but this does not reflect the reality. A venous cannulation which is not perfectly performed can lead to serious life-threatening complications in several steps. The technique we describe step by step is the current gold standard in terms of safety and efficacy: from the use of ultrasound for ultrasound-guided puncture to safe advancement of super stiff guidewires by means of a sentinel catheter, and concluding with smooth insertion of the venous cannula over the stiff guidewire up to the SVC. Moreover, a list of bailout maneuvers to solve complications is presented along with a report of institutional clinical experience since the adoption of this technique.

## Introduction

Peripheral puncture and cannulation of femoral vessels have become common practice in contemporary minimally invasive cardiac surgery (MICS), which requires increasing adoption of guidewires, catheters, and interventional skills (1, 2). If not performed correctly, following an established step-by-step approach, consequences can become fatal complications for patients (iliac or caval veins rupture with massive internal bleeding). The femoral venous puncture is used in many different clinical contexts, from the cannulation to institute the extracorporeal circulation for MICS (mainly valvular), either for the placement of veno-arterial and veno-venous ECMO cannulas and for structural transcatheter interventions in which surgeons are involved (e.g., tricuspid and transseptal procedures) (3). In order to avoid fatal issues with patients, it is essential to know where and how to puncture the femoral vein, how to safely advance the venous cannula up to the superior vena cava, and which precautions to adopt to successfully cope with complications of this procedure. The technique may vary depending on the patient, and every surgeon should know how to adapt the procedure. This study aims to cover the percutaneous puncture of the vein and safe positioning of a two-stage percutaneous venous cannula in the superior vena cava step by step. There will be focus on imaging to visualize the structures under echocardiographic guidance, and the final section is dedicated to troubleshooting.

## Materials and Equipment

- Sterile cover, 7.5-MHz linear ultrasound transducer [Sonosite II; Fujifilm, Japan], and ultrasound gel.
- Transesophageal TEE ultrasound transducer, the echocardiography unit [EPIQ 7, Philips, NL].
- Luer slip syringe 20 ml (not luer lock), 18-gauge needle, blade scalpel.
- Femoral introducer sheath 6 Fr and curved Klemmer forceps to prepare subcutis.
- Vascular dilators for percutaneous cannulation technique: 8, 12, 16, 20, and 24 Fr [LivaNova PLC, United Kingdom].
- Standard Emerald diagnostic J-Tip guidewire 0.035 × 180 cm, Amplatz Super Stiff guidewire or Back-Up Meier J-Tip guidewire of more than 180 cm [Cordis, United States].
- Angiographic diagnostic catheters, pigtail-shaped, 90 cm (e.g., pigtail 5 or 6 Fr)[Cordis, United States].
- Femoral venous cannulas (several models) with a dual stage tip that drains from superior and inferior vena cava, normally available in two sizes (22/22 and 23/25 Fr) [Medtronic, United States; LivaNova PLC, United Kingdom].

## Methods

**Figure 1:**
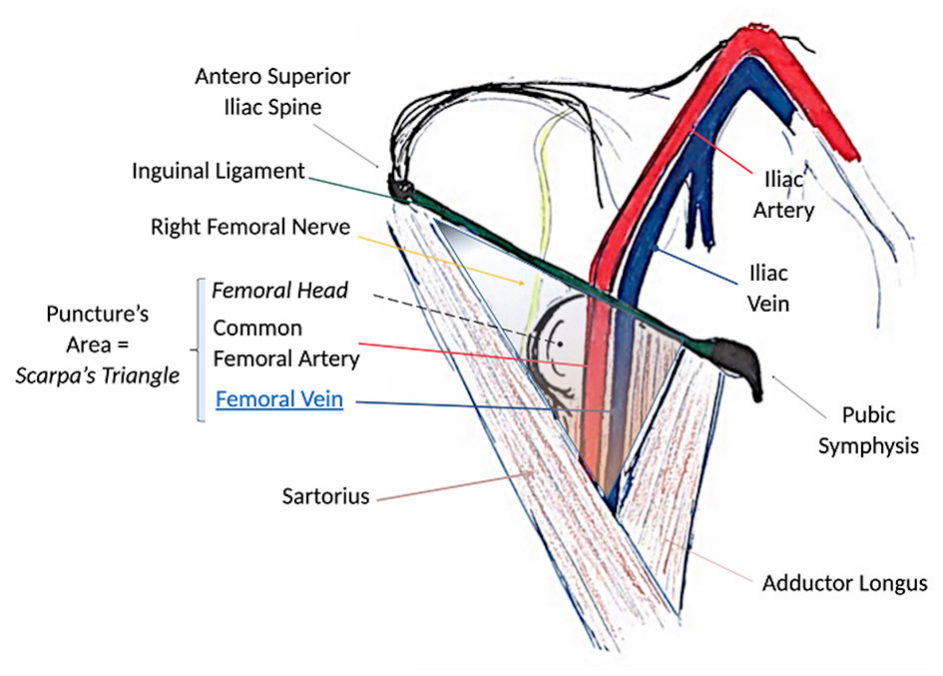
Surgical anatomy of the groin and femoral anatomical structures. Note that the path of the femoral vein is always lying medial to the femoral artery below the ligament. The femoral nerve lies invariably lateral to the artery. Scarpa's triangle is the region of interest for the puncture.

**Figure 2:**
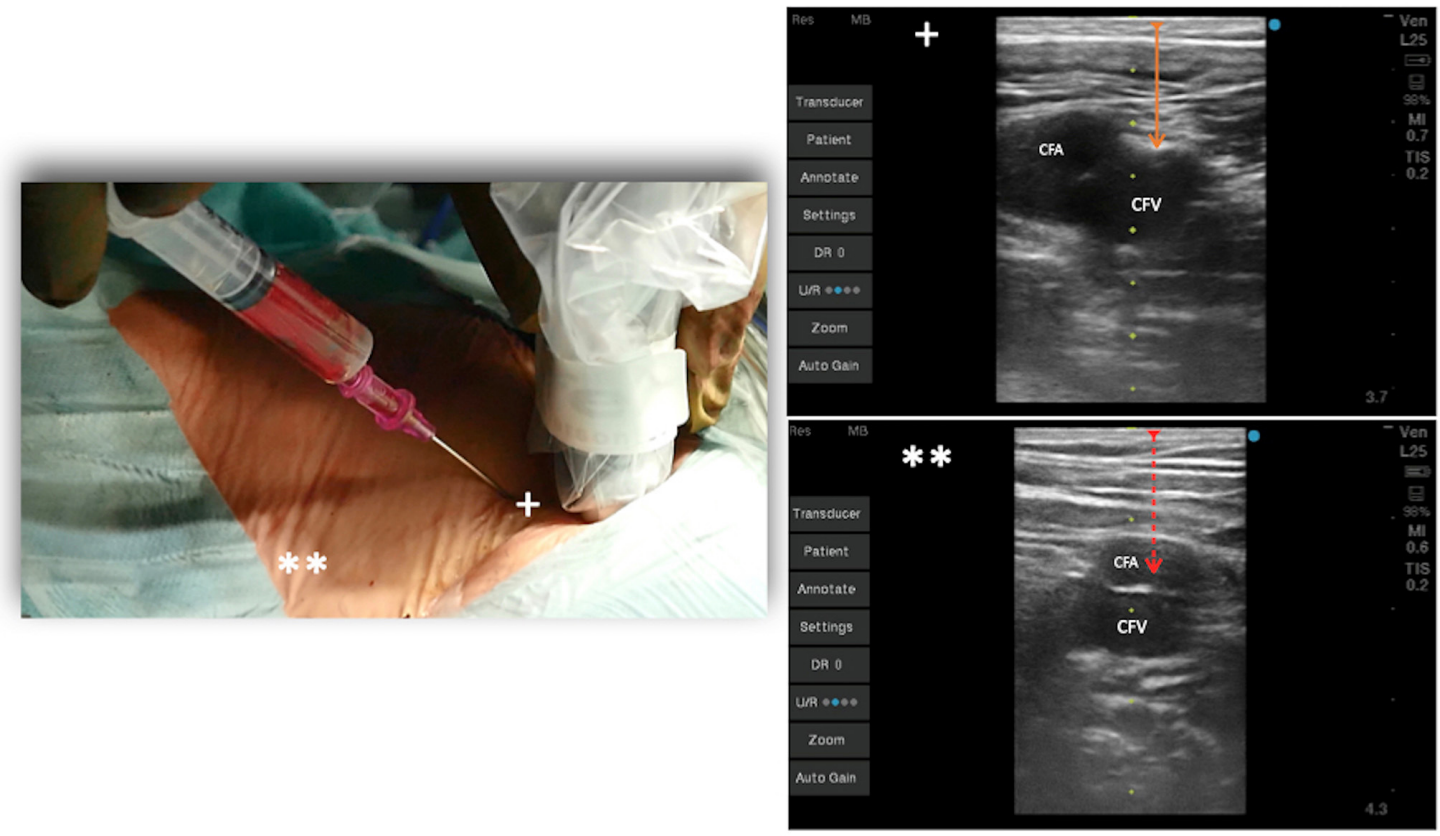
Venous puncture at 45° at the level of the groin under echo guidance. ^+^Vein lateral to the artery, **vein under the femoral artery, distal toward the leg.

Today, to perform an optimal femoral vessel puncture, the essential tool is the real-time ultrasound probe, so a quick angio study on the femoral vein can be performed and the Seldinger technique can be adopted. Consequently, the vein will be punctured at the right spot, which is the one lying below the inguinal ligament and cranial to the saphenous cross (common femoral vein; Figure 1). The puncture of the vein should be performed in an area where it could large enough, and it should not be under the artery (Figure 2). The vein is confirmed by the possibility of compressing it, while the artery is not compressible; for double checking, the color-doppler is a useful tool. A 20-ml luer slip syringe (the luer lock attachment is not recommended in this case, as it does not prevent accidental movements during de-connection from the puncture needle), usually filled with 10 ml hydrosaline solution, and a hypodermic needle were used (Figure 2). The vein is usually punctured 1–2 cm below the groin. It is advisable not to puncture the skin vertically but with an angle of 30° coming medially. In this phase, the operator should avoid damaging the femoral artery; the vein is almost invariably medial to it. Usually, in the first 1–2 cm distal to the groin fold, the course of the vein and artery is well divided, while the vein progressively presents a course exactly below the femoral artery, making the puncture at that point too distal and dangerous for potential arteriovenous fistulas (Figure 2).

**Figure 3:**
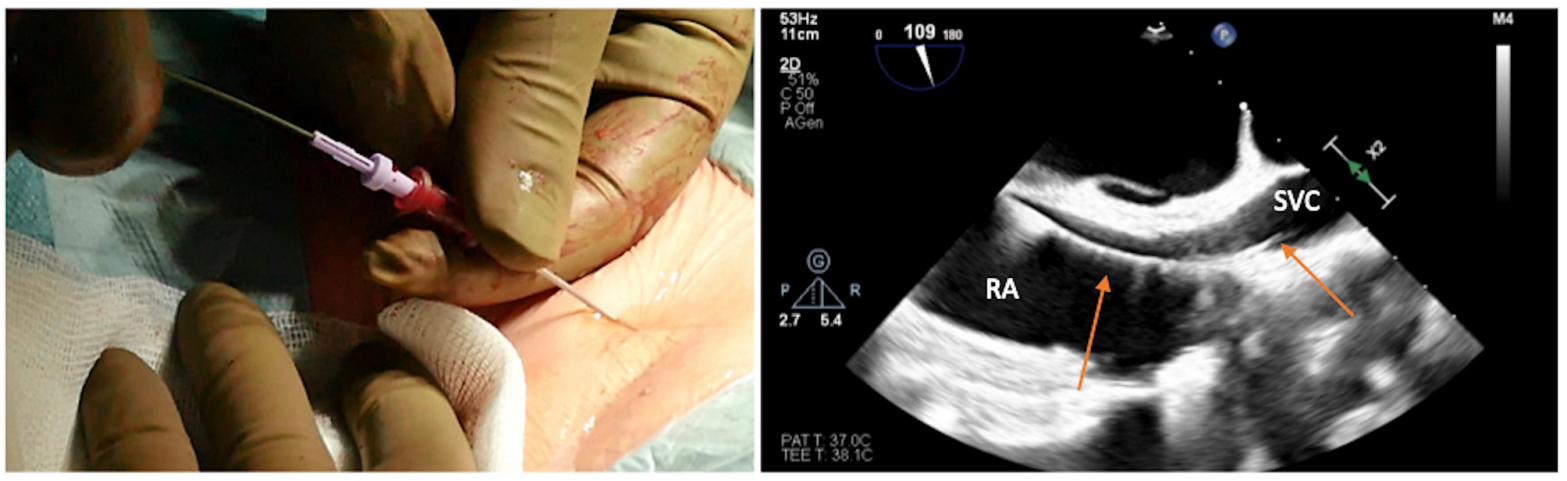
Advancing the soft J-tip guidewire through the needle up to the superior vena cavca (SVC), and visualizing the right atrial (RA)/SVC junction on transesophageal echocardiogram in bicaval projection.

The risk is inadvertently puncturing the artery and passing through it with the needle, side to side, entering the underlying vein. Once the dark, deoxygenated blood is recognized, the syringe is removed from the needle, and a standard 180-cm diagnostic guidewire is inserted, taking care of any resistance (Figure 3).

**Figure 4:**
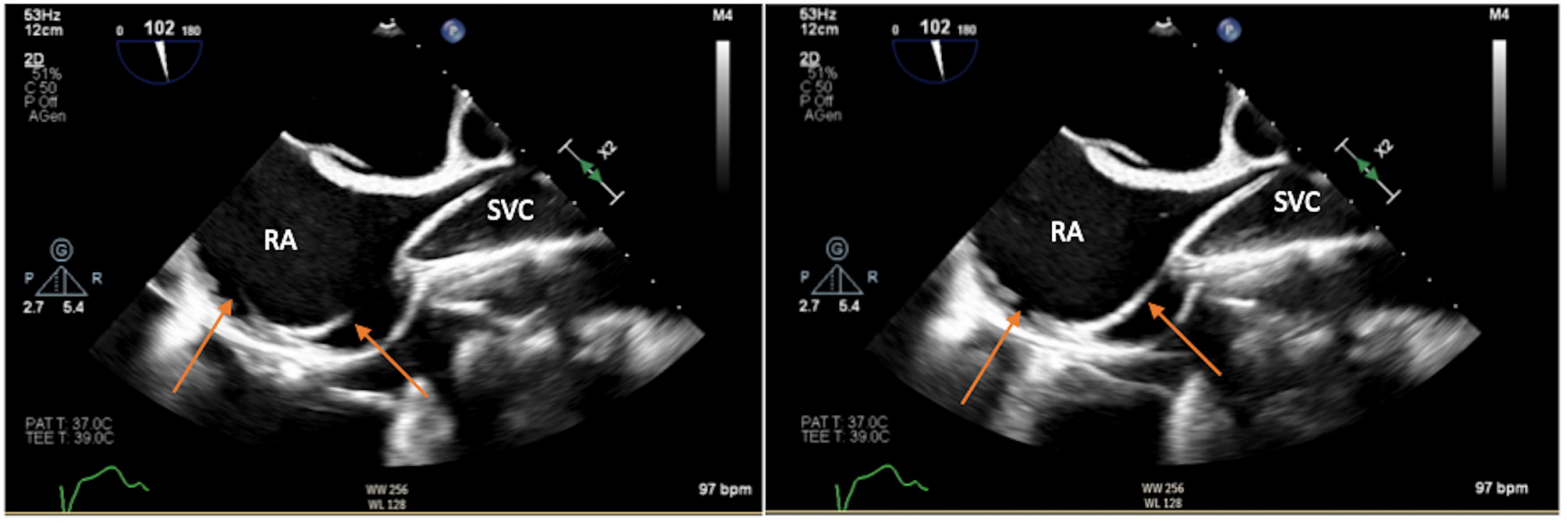
Loops of the soft guidewire in the right atrium, a condition which should be avoided.

The target of the peripheral cannulation is the superior vena cava, 2 cm above the superior cavoatrial junction (Figure 3). This is where the guidewire under transesophageal echocardiographic guidance (bicaval projection) should be positioned and where the tip of the venous cannula will finally land.Visualizing the standard guidewire in the superior cava, without atrial loops (Figure 4), is also a fundamental step, which requires clear and unambiguous communication in the operating room between the cardiac surgeon and the anaesthesiologist or the echocardiographer.

**Figure 5:**
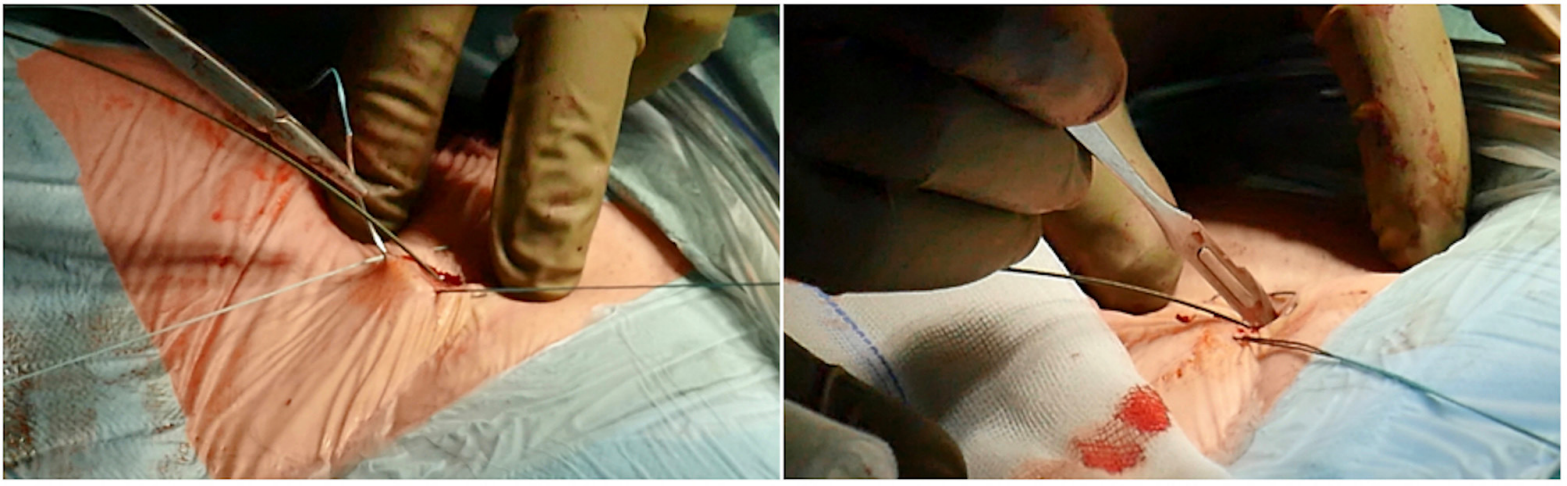
Circular or Z-shaped surgical stitch is prepared with a woven 0-suture to close the access around the cannula and then at the end for removal. On the right, it is essential to prepare the subcutaneous tissue separating the vein from the skin with a scalpel or a curved Mosquito forceps: the vast majority of kinking of wires occurs because of the resistance offered by these connective tissues.

If the room is a modern hybrid one or if you are in a cath-lab, you can perform fluoroscopy to check exactly the course of the wires, and the insertion and final positioning of the cannula. A circular or Z-shaped surgical stitch is prepared with a woven 0-suture to close the access around the cannula and then at the end for removal (Figure 5).

**Figure 6:**
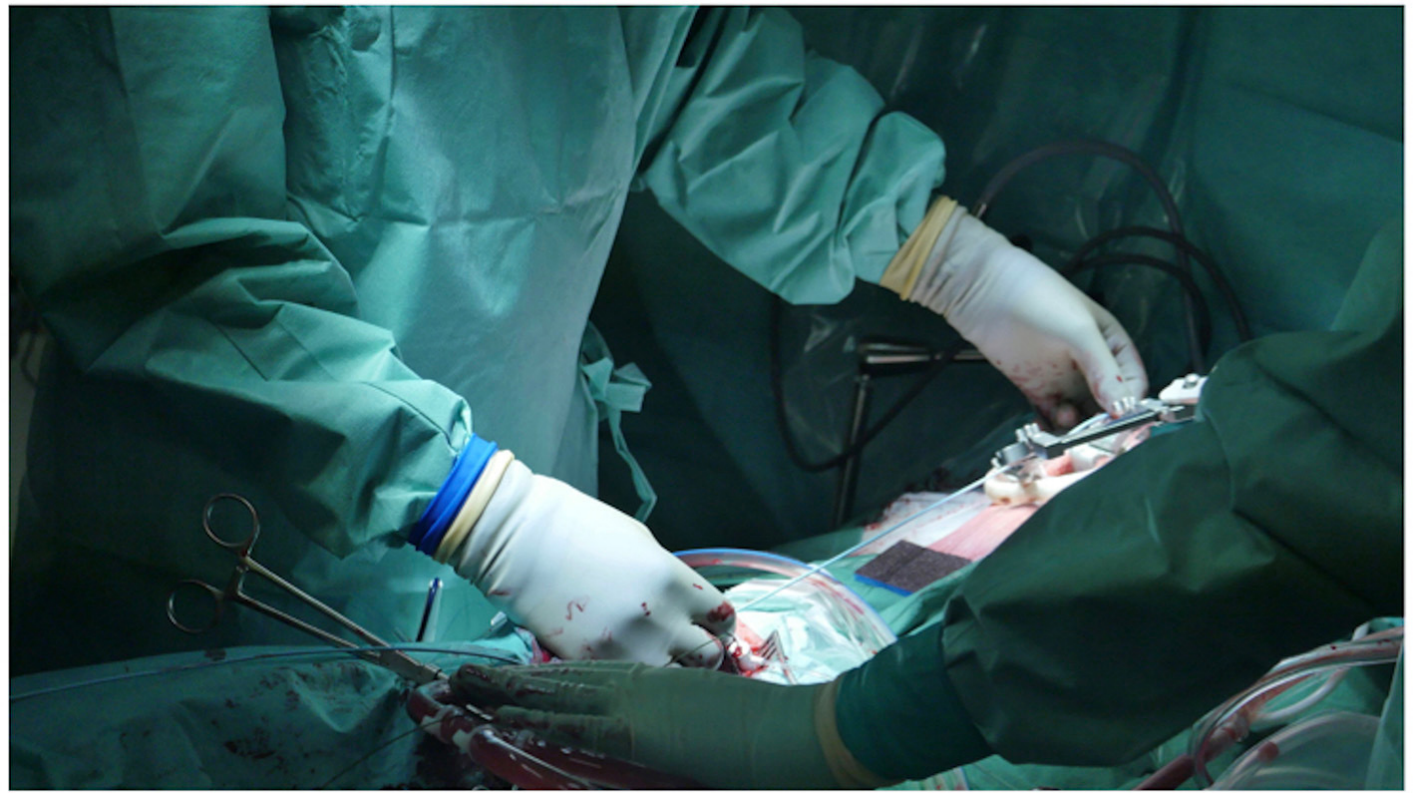
In the absence of fluoroscopy, one can take measurement from the venous access to the SVC externally to the thorax by marking the distance on the catheter with two fingers of the right hand.

**Figure 7:**
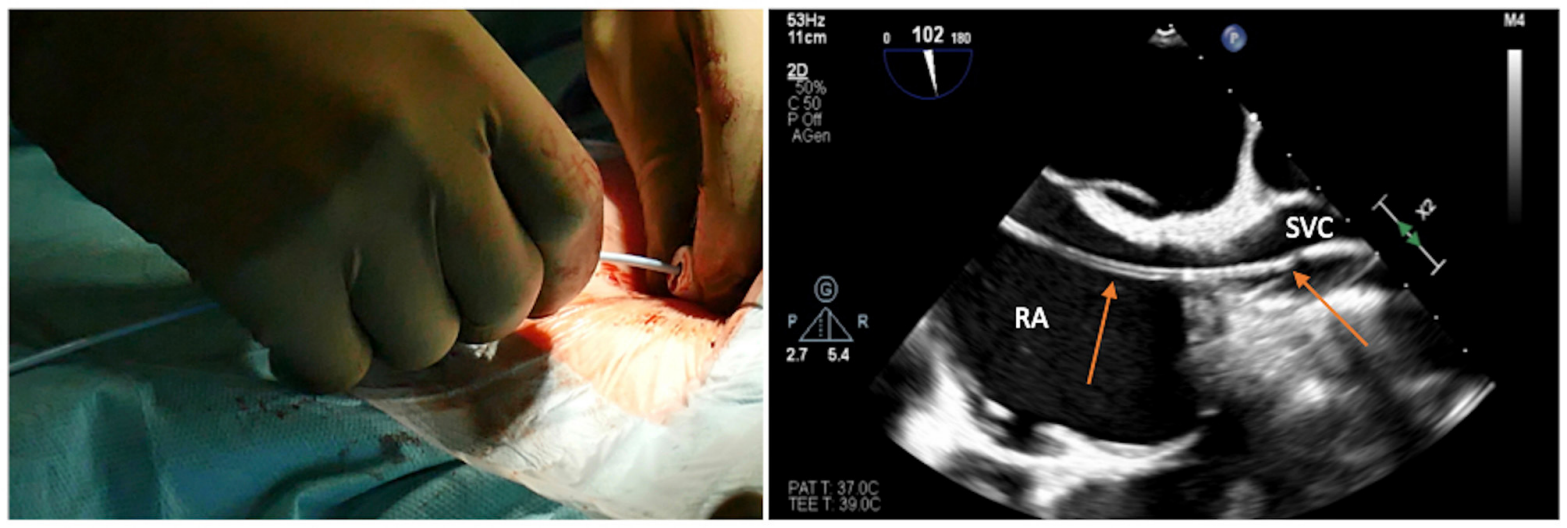
Pigtail is inserted over the standard floppy guidewire up to the SVC; the latter is withdrawn very carefully for the catheter tip to remain positioned in the SVC.

**Figure 8:**
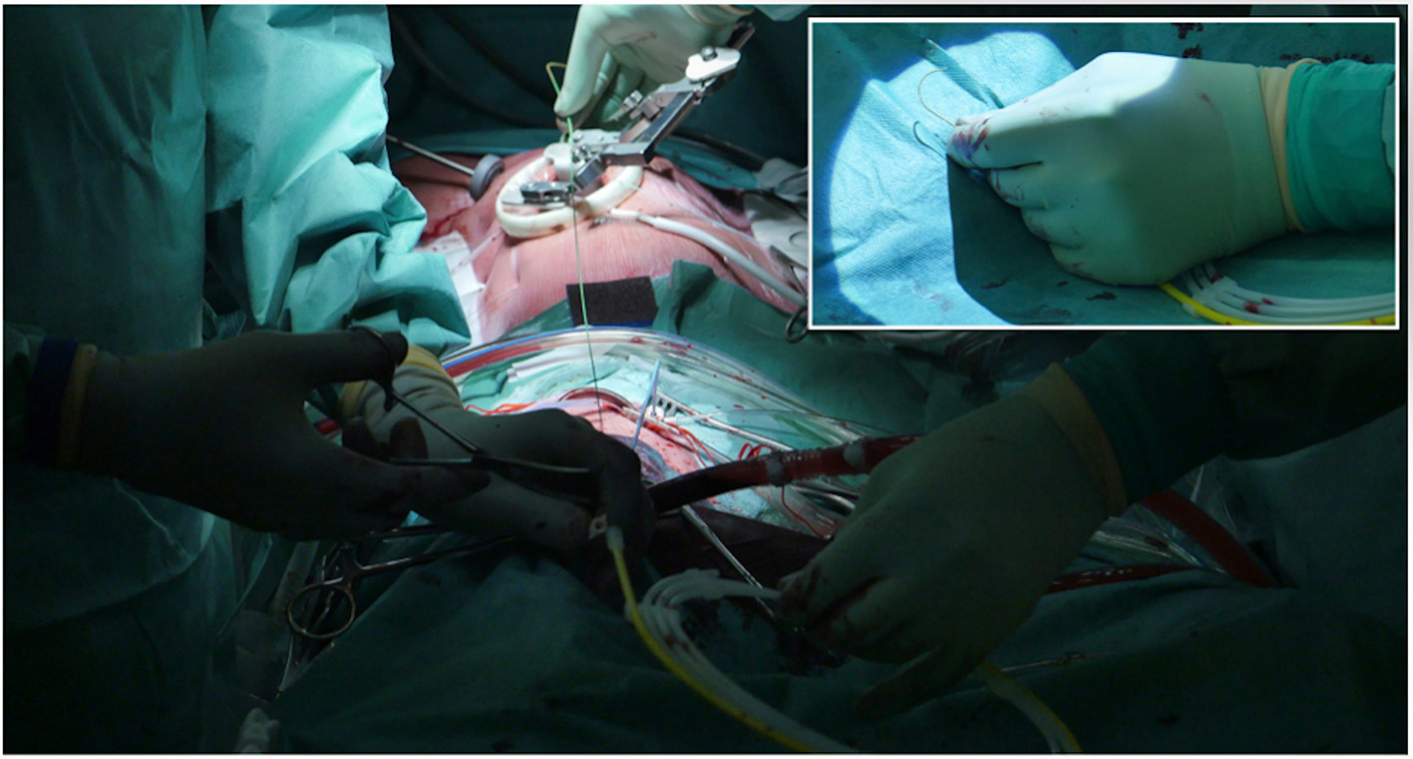
Before inserting the stiff wire, one can calculate the length from the venous access to the SVC externally to the thorax by marking the stiff guidewire with a Klemmer forceps. In the picture in picture, the making of a J at the tip of the stiff.

**Figure 9:**
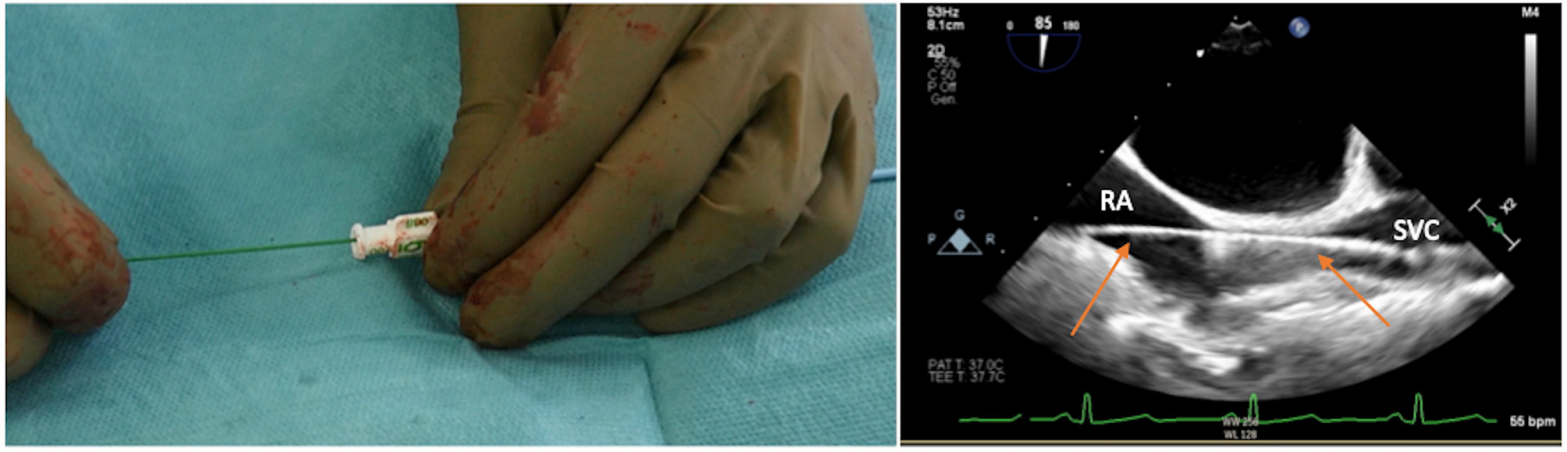
Stiff wire is inserted up to the SVC. In this step, the catheter is removed by exchanging it back on the stiff guidewire, which will remain correctly positioned in the SVC.

**Figure 10:**
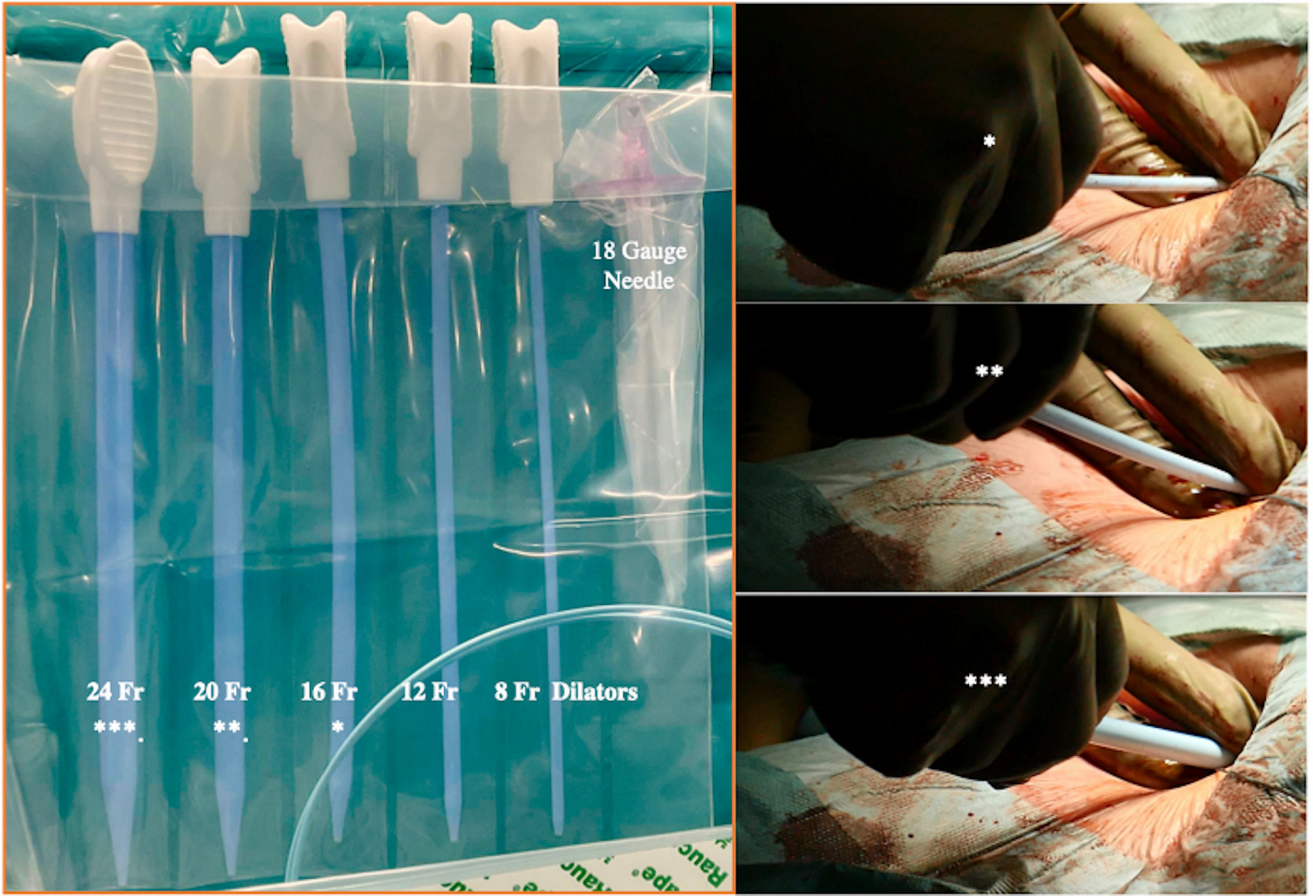
Progressive increase in vascular kit dilator diameter to perform pre-dilatations of femoral vein over the stiff wire (which eliminates dangerous kinking). *In the picture are linking the dilators within the two pictures.

**Figure 11:**
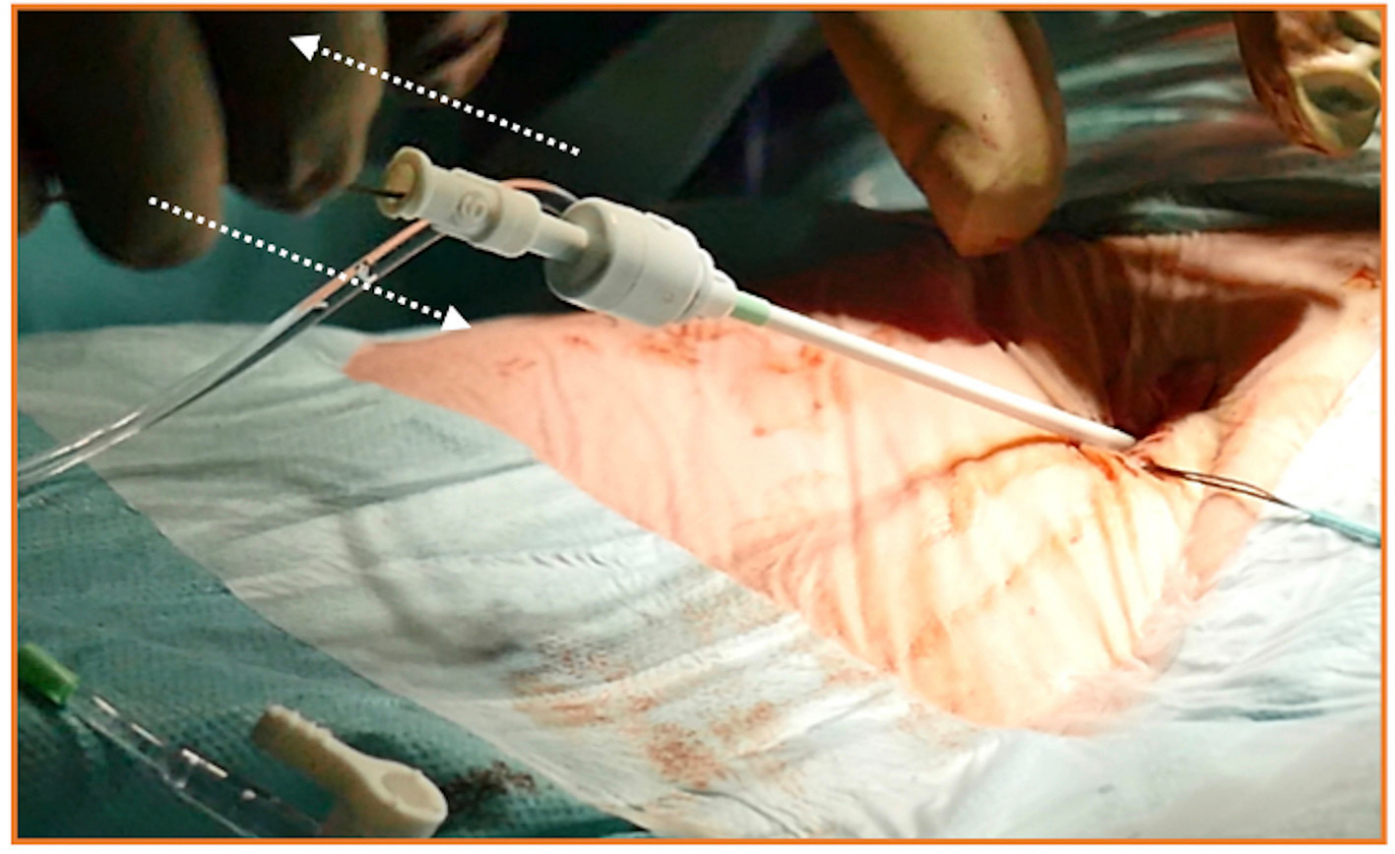
A habit to get into when inserting and exchanging introducers, catheters, vascular dilators, or cannulas, is moving the guidewire slightly back and forth inside them. If a kink is detected or present, it should be promptly detected.

**Figure 12:**
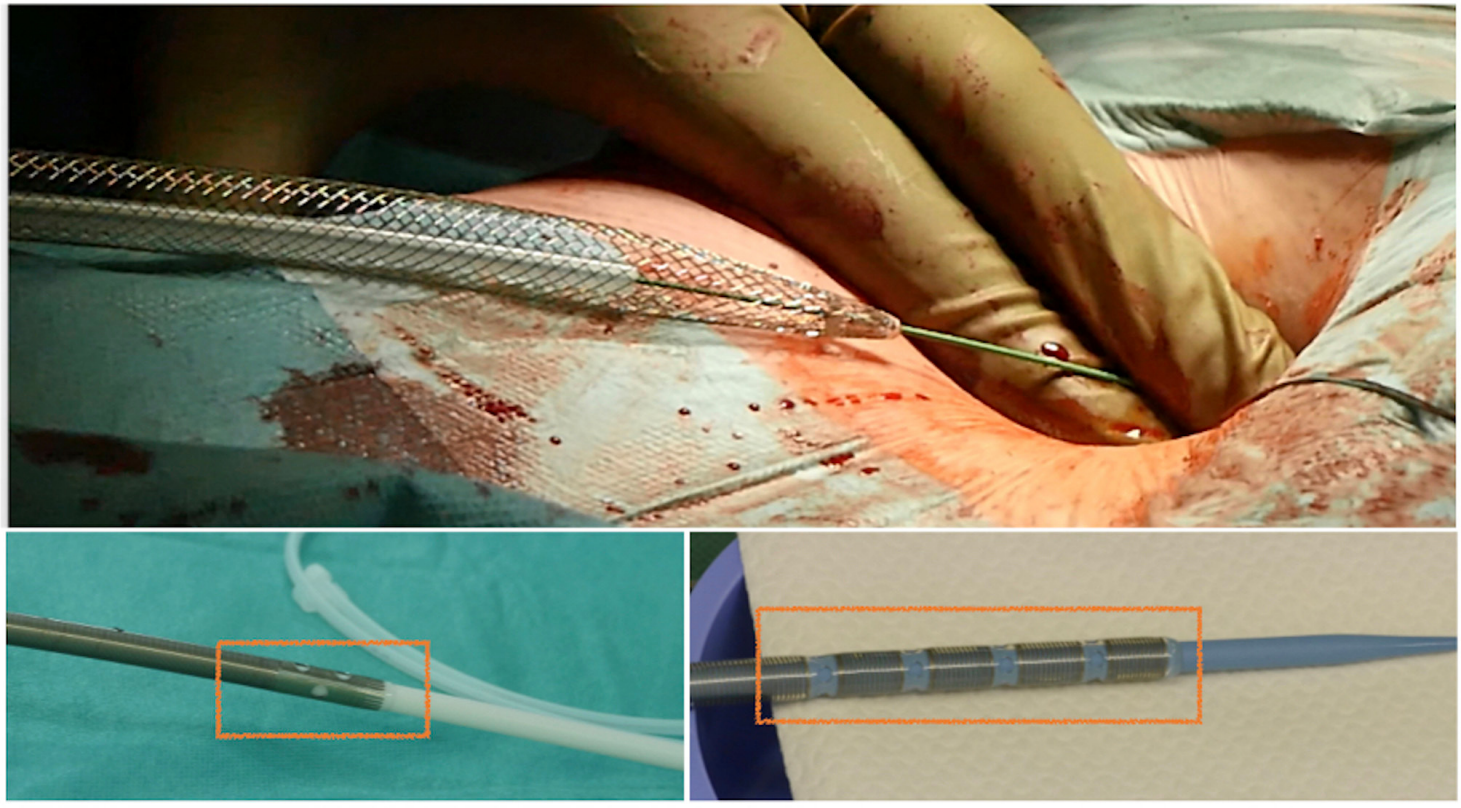
Safe insertion of the venous cannula over the stiff wire, with illustration of several models of single-stage and two-stage venous cannulas for minimally invasive cardiac surgery (MICS).

**Figure 13:**
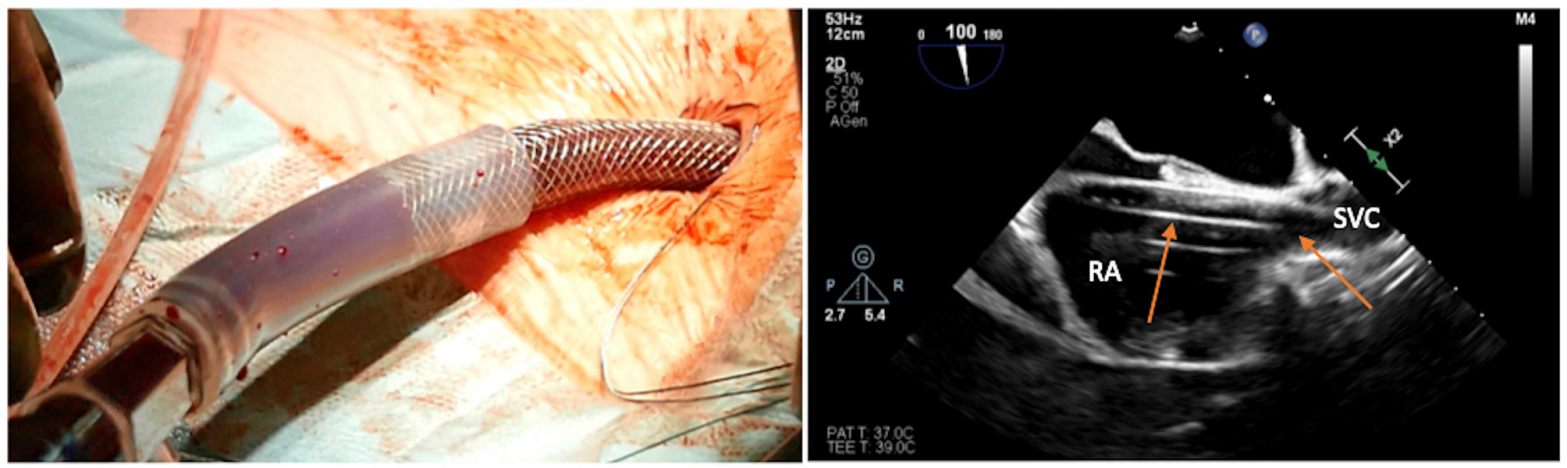
Transesophageal echocardiographic confirmation of positioning in bicaval projection of the tip of the venous cannula in the SVC.

It is essential to prepare the subcutaneous tissue separating the vein from the skin with a scalpel or a curved Mosquito forceps: the vast majority of kinking of the wires occurs because of the resistance offered by these connective tissues (Figure 5). In order to perform the safest venous cannulation, it is, therefore, necessary to go for stiff or super stiff guidewires (e.g., Amplatz Super Stiff guidewire or Back-Up Meier J-Tip Steerable guidewire) of more than 180 cm length. To insert these very stiff guidewires, which offer stability but easily tear the vessels, a 90-cm long sentinel catheter (e.g., Amplatzer Left or pigtail 6 Fr) should be routinely used as protection. In this stage, a therapeutic dosage of unfractionated heparin (UFH) can be administered. The 90-cm length of the angiographic diagnostic catheter is key to avoid perforations when exchanging wires. In the absence of fluoroscopy, one can take the measurement from the venous access to the superior cava externally to the thorax, marking the distance on the catheter with two fingers of the right hand (Figure 6). The pigtail is inserted over the standard floppy guidewire up to the superior cava, and this is later withdrawn very carefully so that the catheter tip remains positioned in the superior cava (Figure 7). Again, before inserting the stiff wire, one can calculate the length from the venous access to the superior cava externally to the thorax, marking the stiff guidewire with a Klemmer forceps (Figure 8). The stiff wire is inserted up to the Klemmer and will be well positioned in the superior vena cava. In this step, the catheter is removed by exchanging it back on the stiff guidewire, which will remain correctly positioned in the superior vena cava (Figure 9). Now, progressive dilation of the venous access is carried out using appropriate dilators (vascular dilator kits of increasing diameter; Figure 10). It is advisable to move the guide wire slightly back and forth inside them: if the wire moves freely, it means that it is not kinked (Figure 11). Of note, the last dilator should have the same size (French catheter scale) as the percutaneous venous cannula intended to be used, not longer to avoid bleeding around the cannula. The dilation procedure is performed at the safest level on the stiff guidewire, since floppy wires are exposed to a frequent risk of kinking and complications, being usually very soft. The dilation process proceeds adopting the dilators in sequence (Figure 10) and the insertion of the venous cannula, with transesophageal echocardiographic confirmation in bicaval projection (Figures 12, 13). In removing the percutaneous cannula introducer, either for a dual stage cannula or a smart cannula, the tip should remain well positioned approximately 2 cm above the superior cavoatrial junction (Figure 13).

## Results

Until September 2020, venous cannulation during MICS was performed in our Institute according to the preference of the operating surgeon, allowing for a certain degree of variability. In particular, one patient experienced serious complications because of kinking of a soft guidewire during pre-dilatation maneuvres of the venous access. Since then, it has been decided to standardize the approach using a stiff wire for pre-dilatation and advancement of the surgical venous cannula, usually a double-stage rigid one or SmartCannula. To eliminate the risks of perforation associated with advancement of stiff wire, exchange of a sentinel catheter placed in the SVC was adopted, making the cannulation technique easy and effective. Since then, 73 MICS patients (25 aortic valve replacements by ministernotomy and 48 mitral repair by right minithoracotomy) have been operated on with this technique, with excellent results in terms of safety profile. Namely, no complications have been reported with this technique apart from minor and very occasional groin hematomas of venous origin treated conservatively by compression. The procedure, double guidewire exchange, takes an average of 5 min longer to perform, but pre-dilatation and cannula insertion are performed with abolished risk of either guidewire kinking or perforation. Explorative surgical cut down of the groin due to bleeding or injury of the vessels has never been required.

## Discussion

The current cardiac surgical landscape, with the expansion of minimally invasive operations, ECMO, and some interventional therapies, requires thorough knowledge of peripheral cannulation techniques. In particular, venous cannulation may appear trivial and complication-free, but this does not reflect the reality. A venous cannulation that is not performed perfectly can lead to serious life-threatening complications in several steps. The technique we have described step-by-step is the current gold standard in terms of safety and efficacy, from the use of ultrasound for ultrasound-guided puncture to the safe advancement of super stiff guidewires by means of a sentinel catheter and concluding with smooth insertion of the venous cannula over the stiff guidewire up to the SVC. Below, a list of bailout maneuvers to solve complications has been inserted.

Facing complications: pitfalls and suggested tips during venous cannulation

• How to suspect an arteriovenous fistula
  If inadvertent puncture of the femoral artery and, thereafter, the femoral vein occurs, a fistula between the artery and the vein will be created. Worse, subsequent dilation with dilators along the guidewire and insertion of the cannula by at least 20 Fr will damage the artery irreparably and make the fistula a surgical emergency during surgery or at the time of decannulation because of massive bleeding on systemic heparinization (usually necessitating replacement of the femoral artery with a vascular graft). This issue should be always ruled out when oxygenated blood, even in a small quantity, is gushing out from the venous groin puncture, once the 6-Fr introducer or the small Fr dilator is removed. Urgent surgical repair/replacement of femoral vessels, according to the injury detected, is mandatory and the only solution.
• Tip
  Always preparing the subcutis with a Mosquito or small curved Klemmer is advisable to avoid kinking of the guidewire during insertion of introducers, catheters, or dilators (Figure 5).
• How to manage resistance
  Feeling resistance to the advancement of the guidewire is one of the things to be most careful about, because most of the time the wire has taken the wrong route and is in the contralateral iliac vein, renal vein, or hepatic vein. Pushing against resistance will easily produce tears, ruptures, and dissections of the vein and have fatal consequences for the patient. In this case, a 6-Fr sheath can be temporarily inserted in the vessel, and the target structure (superior vena cava or descending aorta) can be reached by inserting a pigtail.
• How to avoid kinking a standard guidewire
  Stay parallel to the skin while dilating and do not point and push in an orthogonal manner with respect to the vein.
• How to manage a kinked guidewire
  As previously mentioned, a habit to get into when inserting and exchanging introducers, catheters, vascular dilators, or cannulas is moving the guidewire slightly back and forth inside them (Figure 11). If the wire moves without resistance, it means it is not kinked. If it gets stuck or offers resistance to movement, the wire is kinked and needs to be replaced to avoid further complications. In case of a cannula, a sheath, or a dilator not advancing over a wire, the most common problem is kink. Forcing the advancement of catheters is dangerous and should be avoided. The possible solutions are: pull back the wire a few centimeters until the kink becomes visible and try to advance the catheter/sheath/dilator beyond the kink under direct vision and after manual straightening of the wire (rarely a kinked wire can be successfully straightened). Another alternative solution is to advance over the kinked wire a dilatator of a small sheath (best is a 4 F or a 6F) having care of advancing slowly and concomitantly pulling back the wire a few millimeters. Once the dilator is inside the vessel, the kinked wire can be safely removed and replaced with a new wire in the vessel through the dilator.
• Tip
  Avoid advancing extra stiff guidewires in the veins without the protection of a sentinel catheter because there is high risk of tearing the walls.
• How to manage loops
  If a too much floppy guidewire is inadvertently advanced, it will enter the right atrium and create loops. This happening will make it dangerous to advance both the sentinel catheter and the cannula over the looped wire, and will lead them into a false route (transseptal or to the right ventricle and pulmonary artery) (Figure 4).
• Tip
  The length of pigtail should be 90 cm, and the stiff guide (backup Meier or classic stiff) should be at least 180 cm, ideally 210 cm, to avoid conflicts at the moment of exchange when the guide is finished and the pigtail is not yet out of the femoral vein.

### Simulators for Venous Percutaneous Cannulation Procedures

**Figure 14:**
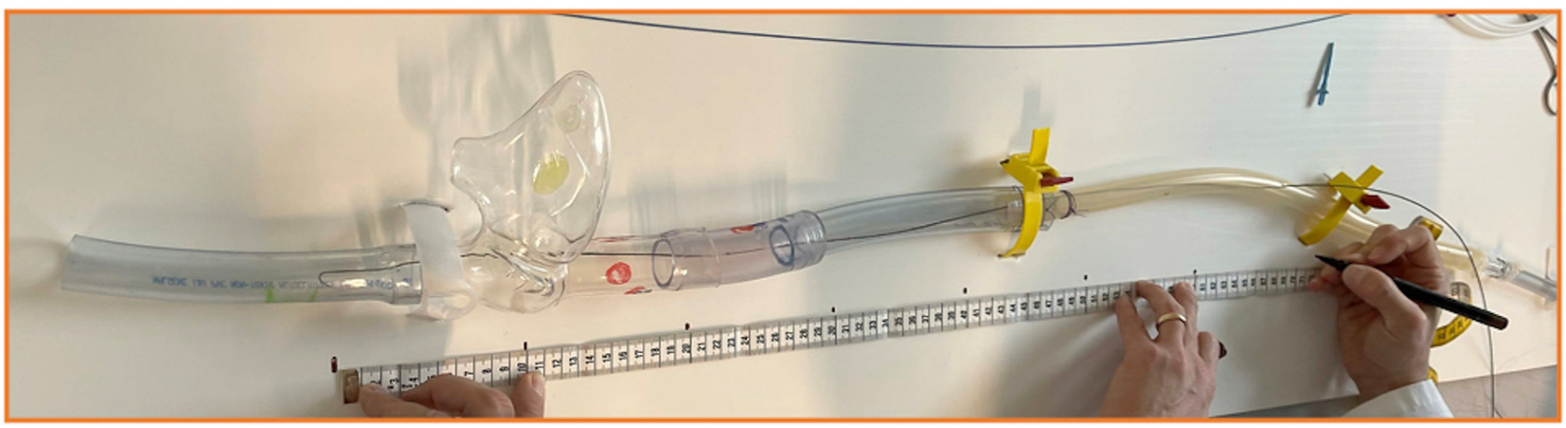
Low-fidelity simulator to train and experience all the steps of the cannulation procedure.

Simulation has become integral to the training, either for medical students or medical professionals. In order to understand what really happens during the cannulation process and how the exchange of guidewires through the sentinel catheter takes place, low-fidelity models can be used to mimic the anatomical distance and exercise the procedure on the bench. A focus on the superior vena cava makes it possible to calculate very well what material and length of guidewires and catheters are need (Figure 14). We established a simulation model composed of a synthetic material that provided an inexpensive low-fidelity model. The simulation component represents the venous system from the femoral to the SVC, in order to be punctured and perform the basics of the procedure adopting the right movements and materials. Low-fidelity simulators are extremely inexpensive and easily assembled, allowing team members to develop, master, and maintain interventional skills necessary for percutaneous venous femoral cannulation (Supplementary Video 1).

## Data Availability Statement

The original contributions presented in the study are included in the article/Supplementary Material, further inquiries can be directed to the corresponding author.

## Author Contributions

AP, TTo, EF, and SD provided substantial contributions to the conception or design of the work, the acquisition, analysis or interpretation of data for the work, and drafted the work or revising it critically for important intellectual content. FT and TTh provided approval for publication of the content. All authors agreed to be accountable for all aspects of the work in ensuring that questions related to the accuracy or integrity of any part of the work are appropriately investigated and resolved. All authors contributed to the article and approved the submitted version.

## Conflict of Interest

The authors declare that the research was conducted in the absence of any commercial or financial relationships that could be construed as a potential conflict of interest.

## Publisher's Note

All claims expressed in this article are solely those of the authors and do not necessarily represent those of their affiliated organizations, or those of the publisher, the editors and the reviewers. Any product that may be evaluated in this article, or claim that may be made by its manufacturer, is not guaranteed or endorsed by the publisher.
